# Biotechnical Control of *Varroa* in Honey Bee Colonies: A Trade-Off between Sustainable Beekeeping and Profitability?

**DOI:** 10.3390/insects14100830

**Published:** 2023-10-23

**Authors:** Monica Vercelli, Luca Croce, Teresina Mancuso

**Affiliations:** 1 Independent Researcher, 10133 Turin, Turin, Italy; 2 Independent Researcher, Borgata Baratta 27, 10040 Villardora, Turin, Italy; 3 Department of Agricultural, Forest and Food Sciences (DISAFA), University of Turin, Largo P. Braccini 2, 10095 Grugliasco, Turin, Italy; teresina.mancuso@unito.it

**Keywords:** beekeeping farm, economic, profitability, biotechniques, *Varroa* control, honey bees, *Apis mellifera*, sustainability, best management practices

## Abstract

**Simple Summary:**

Beekeeping is an important economic activity but the sector suffers from both technical and economic problems. One of the main threats affecting honey bees is the *Varroa destructor* mite. To improve beekeeping farm sustainability, low-environmental-impact control of this mite is recommended. This paper examines different methods applied for the control of *Varroa* on Italian beekeeping farms both in terms of operations carried out in apiaries and net income, focusing on biotechniques. First, our analyses provide a detailed overview of each biotechnique and the organic and conventional treatments used to fight *Varroa*. Then, by highlighting the higher net income of farms applying biotechniques, we show that a trade-off need not be made between sustainable techniques and farm profitability. It is possible to achieve a long-term reduction in *Varroa* infestation while obtaining zero-residue bee products and respecting environmental and human health in compliance with European and national regulations and guidance.

**Abstract:**

Beekeeping faces several challenges, such as the *Varroa* mite. Few studies have measured the economic performance of farms in relation to the practices used for *Varroa* control. Our study analyzed various biotechniques (total brood removal, TBR; queen caging, QC; royal cell insertion, CI) and other methods (chemical treatments, CT; thymol use, THY) adopted by Italian beekeepers to show whether the adoption of biotechniques leads to farm profitability or a necessary trade-off between sustainability and profitability. Beekeepers were interviewed about the methods and operations conducted on their farms. The net incomes (NIs) of the farms were calculated and inter- and intrafarm comparisons were performed. A detailed schema of each practice was designed. The net income derived from TBR was the highest in eight out of the nine case studies, followed by CI and then QC. The NI calculated for farms using CT was lower than that for farms using other methods in two of the case studies. We also analyzed different biotechniques applied by the same farm and found that the NI resulting from TBR was higher than that achieved from the use of QC and CI. Our study suggests that use of biotechniques represents a long-term sustainable solution for reducing the level of *Varroa* infestation, which affects farm net income.

## 1. Introduction

Beekeeping is an economic activity that provides bee products (honey, pollen, royal jelly, propolis and wax), livestock (artificial swarms, packed bees and queen bees) and pollination ecosystem services, which are fundamental to agricultural production and biodiversity conservation [1,2,3,4,5,6]. Other services supported by honey bees and beekeeping are environmental pollution monitoring, apitherapy, api-tourism and social and cultural values [7,8,9,10]. In recent decades, the beekeeping sector has suffered as a result of technical and economic issues, including colony management and losses, and productivity [11,12,13,14]. Multiple factors affect honey bee colonies, such as pests and diseases, the use of pesticides, and climate change [12,15,16,17,18,19,20]. The ectoparasite mite *Varroa destructor* Anderson and Trueman, which is dispersed worldwide, is currently considered the major threat for apiculture honey bees (*Apis mellifera* L.) because it weakens colonies by vectoring a set of viruses and inducing colony losses [21,22,23,24].

Some authors have quantified the economic impact of the decline of honey bee colonies, together with the losses of other pollinators (e.g., wild bees and butterflies), on world agriculture and on the production of honey and other bee products [2,3,4,25].

Management by beekeepers is crucial for the health, overwintering success and productivity of honey bee colonies. Of the technical management issues, *Varroa* control practices are a challenge at the beehive, apiary and farm level because the efficacy of the measures is closely linked to the health and survival of honey bee colonies and, consequently, to the production and profitability of the farm [26].

Beekeepers employ different methods to combat *Varroa* and have carried out many trials in collaboration with researchers to monitor *Varroa* infestation levels and evaluate the efficacy of the controls [27,28,29,30,31].

Measures to control mite populations are used once or several times during the bee season and in combination with each other. The most widely used products are synthetic acaricides that differ according to the country or region of the world [32,33,34]. These chemicals used to kill mites can also have toxic side effects on honey bees and entire colonies and lead to risks related to residues within bee products and the development of resistance [35,36,37,38,39]. Furthermore, most acaricides do not fight the mite inside the brood cells [14].

Recently, there has been a trend in apiculture to move towards an increasingly sustainable farming system [40,41]. Authors have highlighted that the sustainability of the bee farming system is a central challenge for the health of honey bees, humans and the environment, as well as for the economic benefits of the farm [40,41]. One of the objectives of sustainable beekeeping, emphasized by Integrated Pest Management (IPM), Good Beekeeping Practices (GBPs) and Biosecurity Measures in Beekeeping (BMBs), is to remove or at least reduce the dependence on synthetic acaricides by applying them at their minimum effective dose to minimize the accumulation of chemical residues in hive products and to avoid resistance to acaricides [42]. In organic beekeeping, no synthetic acaricides are allowed, as indicated by regulations for the production.

Because alternatives to in-hive chemicals are preferred in the context of sustainable beekeeping, biological and biotechnical practices are proving to be useful tools to keep the mite populations under control [23,27,43,44,45]. In particular, the biotechnique of artificial brood interruption, combined with treatment, enables the greatest control efficacy to be achieved, limiting *Varroa* population growth [46,47,48].

Biotechniques include queen caging, drone brood removal, total brood removal and trapping comb [44,49]. Other research demonstrated that *Varroa* mite control was more effective when broodless conditions were created, either by total removal of brood or caging of the queen, in combination with applications of oxalic acid (OA) or other products [50,51]. Recently, Büchler et al. [52] showed that the application of biotechniques significantly contributed to efficient *Varroa* control and the production of safe bee products. A recent review pointed out that biotechniques are one of the three pillars of IPM [53]. For this study, only one economic analysis of the cost/benefit of implementing *Varroa* control was found. In another recent study by Tubene et al. [54], an economic investigation was conducted to compare the management performance of Best Management Practice (BMP) with Average Management Practice (AMP). The study showed that if the beekeeper (from a small beekeeping farm) sustained BMP for a sufficiently long period of time, their initial investment could be profitable. The BMP involves sustainable operations that disadvantage *Varroa* infestations.

The impact of *Varroa* control methods on beekeeping farm profitability is often overlooked. The cost of *Varroa* control is not always solely related to the cost of acaricide treatments, but can also include the various methods implemented in the apiaries, e.g., in terms of human resources. Furthermore, there is an increase in total income resulting from the production of new nuclei, and more research on this issue is needed. In addition, the advantages resulting from adopting sustainable techniques to combat the *Varroa* mite include the reduced impact on the health of honey bees, humans and the environment, as well as the production of quality hive products which can be sold at a higher price, e.g., organic products.

Information on the profitability and productivity of beehives is important to enable beekeepers to select appropriate *Varroa* control techniques. Net income is the result of management of the beekeeping production process, which includes, as stated by Al-Ghamdi et al. [55], “queen quality and age, ecological conditions, floral composition, colony strength, swarming of colonies, types of hives used and honeybee management practices” [56,57,58]. In addition to all these elements, the type of biotechniques chosen for *Varroa* control will also determine the economic result. To date, some studies have focused on the economic features of bee farms [59,60], but few of these measured the economic results of the farms according to the *Varroa* control practices used [61,62]. In addition, there has been no analysis conducted with regard to how each biotechnique should be carried out during the year, together with a description of the operations to be carried out during each period.

In this context, the aim of our study, under the Interreg Alcotra Project “INNOV’API”, was to assess, at the bee farm level, (i) the different operations carried out by beekeepers in relation to *Varroa* control techniques; (ii) the net income depending on the *Varroa* control methods; and (iii) the economic and environmental sustainability trade-off that results from applying biotechniques compared with conventional techniques.

## 2. Materials and Methods

This study was carried out in the Piedmont region (NW Italy). Italian beekeepers keep a total of 1,782,105 beehives and nuclei. Of the total number of hives, 79% are operated by professional beekeepers who raise bees for income. The region with the largest number of hives is Piedmont with 201,151 hives (12.8% of the national total) of which 89.3% are hives held by farms for commercial purposes, in line with the national trend [63]. The estimated Italian honey production for 2022 is 23,000 t and Piedmont recorded a production of 404,829 t [63].

We selected nine beekeeping farms located in Turin and Cuneo provinces, within the cross-border territories of the Interreg project, using different methods to control *Varroa* mite, including biotechniques. Three farms applied a combination of total brood removal (TBR), queen caging (QC) and old queen replacement by royal cell insertion (CI), one farm had a combination of TBR and QC, and one farm combined TBR and the use of thymol (THY). One farm used TBR alone and two farms used synthetic chemical treatment (CT). The farms were characterized by professional activity and migratory beekeeping (Table 1). Some of them were in organic regimes. Other characteristics of the experimental farm are shown in our previous studies [61,62].

This paper documents the results of our research study, which quantified the costs and revenues obtained by Italian beekeeping farms using different methods, both conventional and organic, to combat *Varroa*. Our previous works identified production costs and revenues with reference to a 1-year period (03/01/n–02/28/n+1). In particular, production costs and revenues were defined at the level of a single hive treated with a specific biotechnique [61,62].

The data collected showed that the beekeeping farms that were analyzed used multiple methods for *Varroa* control. Interfarm (between farms) and intrafarm (within a farm) comparisons were performed. The intrafarm comparisons were of particular significance with regard to the objective of this work. In particular, it is worth emphasizing that the determination of parameters for beehives on farms where some beehives were managed using one technique and others using other techniques allowed rigorous comparisons to be made.

Case study numbers 1, 2 and 4 adopted both the brood removal technique (TBR) with queen caging (QC) or royal cell insertion (CI); farm number 3 adopted the QC technique in addition to TBR, whereas farm number 5 used TBR and Api Life Var (THY). Farm number 6 practiced TBR alone. Farms numbers 7, 8 and 9 applied chemical treatments (CT) only, hence, for these farms, intrafarm comparison was not possible.

The questions to be answered by the study were as follows: (1) does implementation of sustainable practices on a beekeeping farm require higher labor and material costs; (2) does use of biotechniques result in zero-residue products that can be marketed at a higher price; and (3) is it necessary to compromise on the health of products to save costs?

A survey in all beekeeping farms was carried out. The beekeepers were interviewed about the apiary management carried out, with reference to the chosen techniques for *Varroa* control and the relative periods of their execution, taking the treatment period (June–July) as the starting point, and proceeding to the overwintering period (January/February). The result of this survey is summarized in a scheme. We also asked the beekeepers many questions, such as: how each technique was carried out (e.g., operations, materials, time), which intrafarm methods they preferred and why, and if they collected and analyzed honey samples to detect residues. Their responses helped us have an overview of the analyzed case studies.

To evaluate the profitability of the surveyed bee farms associated with the use of certain techniques, their net income was calculated. The data were collected a posteriori via interview with the beekeepers at the end of the analyzed year.

The net income (NI) represents the earnings of the beekeeper or owner of the beekeeping farm who, in addition to their organizational role, provides other productive factors such as labor and capital assets. NI is obtained by subtracting the production costs from the revenue [61], and these are configured differently according to the way in which the farm is managed. In the case studies examined in this analysis, there were two possible situations, one in which the beekeeper provided all aspects of the production process (see Equation (1)), and the other in which the beekeeper made use of salaried labor (Equation (2)).

Net Income = Revenue − (Expenses + Quotas + Tributes)(1)

NI represents the remuneration due to the beekeeper who provides all the inputs and assets, and where revenues and expenses have been calculated as detailed in Mancuso et al. [61] for the nine beekeeping farms.

Revenue (called also gross marketable output value) is the active part of the balance sheet and is composed of products sold, self-consumed or sold to third parties for payment [61], the Honey Bee Colony Inventory (HBCI) [62] and stock products when the values are positive. For each biotechnique (such as total brood removal, queen caging and royal cell insertion) and chemical technique, total revenue includes honey and other bee products (i.e., wax, pollen, propolis, nuclei, queens, royal cells, stocks).

The biotechniques differed in terms of expense items and the following expenses were recorded and compared: combs and/or frames with wax, feeding, treatments, royal cells and queens, losses of honey bee stocks, and other expenses [62]. Quotas represent the cost of amortization of owned fixed assets, together with their insurance and maintenance. Tributes are costs incurred according to the tax system of the country and include taxes and fees.

The second situation differs only where part or all of the work (labor) is carried out by external workers, i.e., waged workers:

Net Income = Revenues − (Expenses + Quotas + Tributes + Labour)(2)

The net income is calculated based on all cost and revenue items obtained by the farm in a calendar year, or with reference to a 12-month period for beekeeping farms which ranges from 03/01/n to 02/28/n+1. The net income can then be related to a hive, or group of hives, to which one or more biotechniques were applied. For the farms that applied only chemical treatments to their colonies, the NI is the difference between the costs and revenues for all the bee stock.

## 3. Results

**Figure 1:**
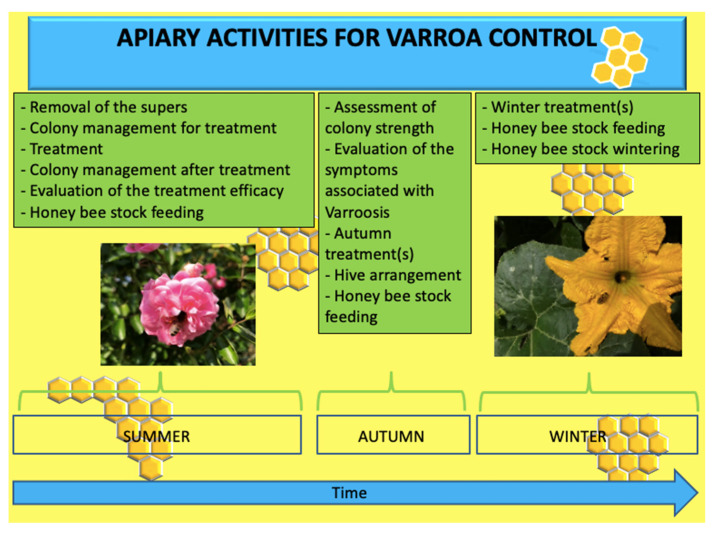
List of activities carried out in the apiary for *Varroa* control (from June to February).

The finding from the interviews of the beekeepers gathered information on all the operations conducted in the apiary, from the preparation of the *Varroa* control treatment to the end of the bee season. The main activities conducted in the apiary in summer, autumn and winter, as reported by beekeepers, are summarized in Figure 1.

The interviews revealed that some operations are common to all types of *Varroa* control, such as removal of the supers, assessment of colony health and evaluation of colony strength consisting in the estimation of the presence of the queen, the number of adult bees, the surface occupied by brood, and by stocks of honey and pollen. In recent years, this type of monitoring has also been conducted using new tools [39].

Beekeepers also reported that some *Varroa* control techniques require more specific attention, precision and manual skill than others. For each method applied, an assessment of the infestation rate was always made before treatment, followed by an assessment of *Varroa* fall on the hive bottom after each treatment. Biotechniques included the use of oxalic acid (OA). The OA was administered using the trickle method (Api-Bioxal, Chemical Life S.p.a., 6.2% oxalic acid dihydrate) at a dose of 5 cc/comb covered by honey bees, whereas the OA acid applied by sublimation was used at a dose of 2 g/colony. One farm used thymol, and the two farms applying synthetic chemical treatments adopted Amitraz or Fluvalinate. All the treatments were carried out in accordance with the rules of the Italian veterinary authorities (use of regular sanitary treatments and a deadline of 15 August).

**Figure 2:**
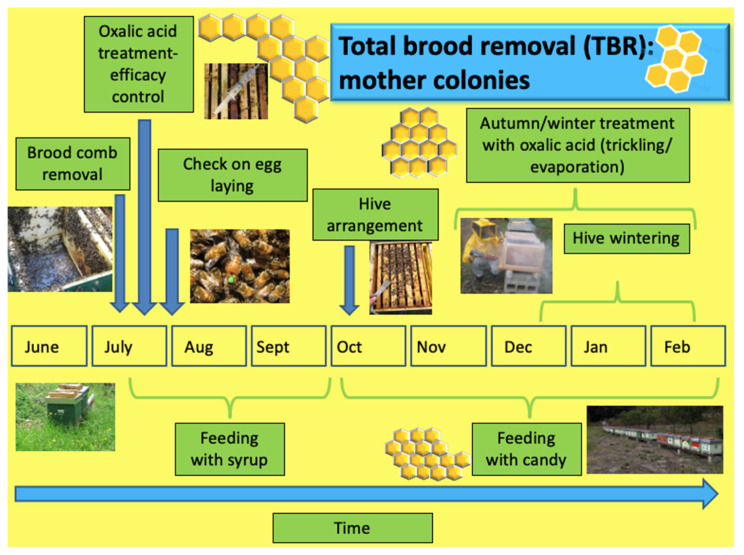
Schema of the operations carried out in the apiary on mother colonies for the total brood removal technique.

**Figure 3:**
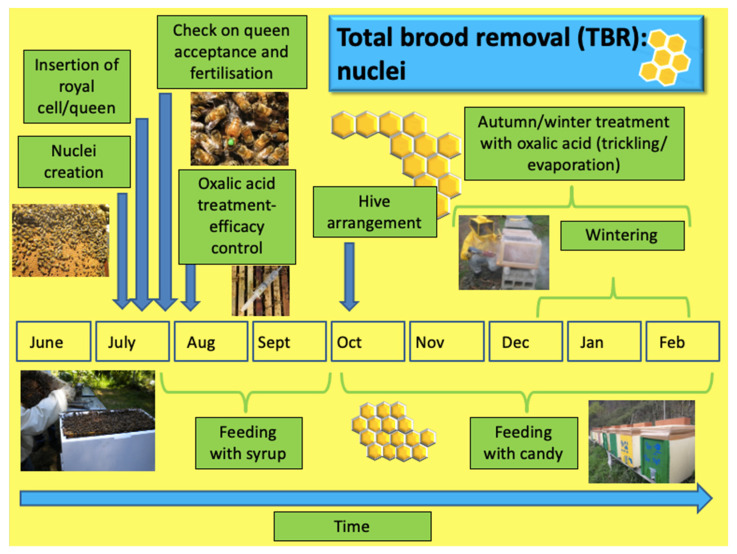
Schema of the total brood removal technique involving the production of the nuclei and related operations in the apiary.

With regard to TBR (Figure 2 and Figure 3), the beekeepers explained that the first step of this technique involves the total removal of the brood from the mother hive to artificially create a broodless colony. This is achieved by moving the combs containing the brood to a nucleus hive with some worker bees. To restore the removed combs, a new few frames or combs are inserted. The mother hive, containing most of the original bees, the queen, and the combs with honey and pollen are treated the following day with oxalic acid to kill *Varroa* in the phoretic stage. After a few days, the queen and eggs are checked for possible failure due to the treatment. Where needed, sugar nutrition by syrup is used.

A royal cell or a fertilized queen is inserted into the formed nucleus. Alternatively, the honey bees are allowed to raise a queen from the present brood. Treatment with dripped OA is administered twice, on the seventh and twenty-first day after nucleus formation. After an interval, which depends on the choice made regarding the methodology adopted to obtain an egg-laying queen, a laying check is carried out. If a new queen is not present or has not laid eggs, the orphan nucleus can be reunited with a nucleus with an active queen or a new queen can be inserted.

**Figure 4:**
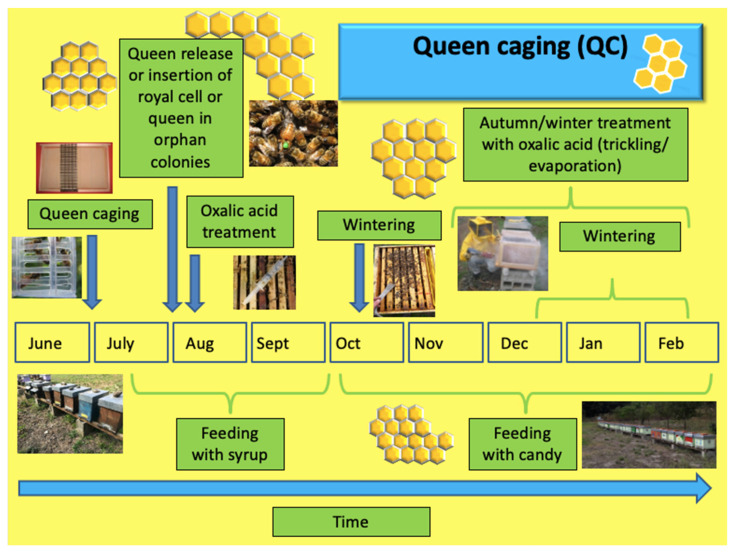
Schema of the queen caging technique and related operations in the apiary.

**Figure 5:**
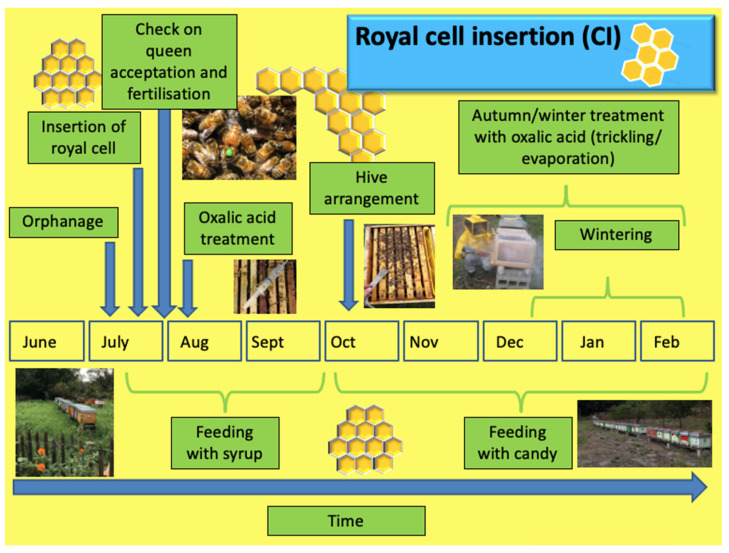
Schema describing the operations for the royal cell insertion technique.

QC and CI techniques (Figure 4 and Figure 5) were found by beekeepers to be less labor-intensive than TBR. QC involves caging the queen between June and July so as to artificially block egg-laying. Cages or modified frames are used for this purpose. After 24 to 25 days, the queen is released and treatment with dripped oxalic acid is carried out. In case of orphanity, a royal cell or fertilized queen is inserted.

For CI, the first step is the orphanage of the colony to achieve two simultaneous results: the lack of a brood and the presence of a new queen meeting certain parameters according to the needs of the beekeeper. The process is carried out in July and is followed by the insertion of a royal cell. After 24 to 25 days to allow the previous brood to emerge and have *Varroa* in the phoretic phase, the fertility of the queen is checked by observation of the eggs and then treatment with dripped oxalic acid is carried out.

**Figure 6:**
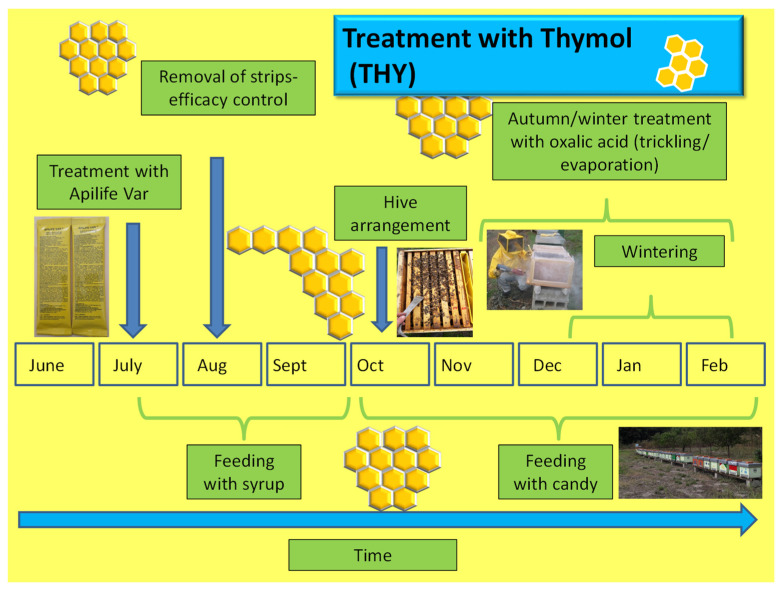
Schema of thymol application and other operations carried out in the apiary.

**Figure 7:**
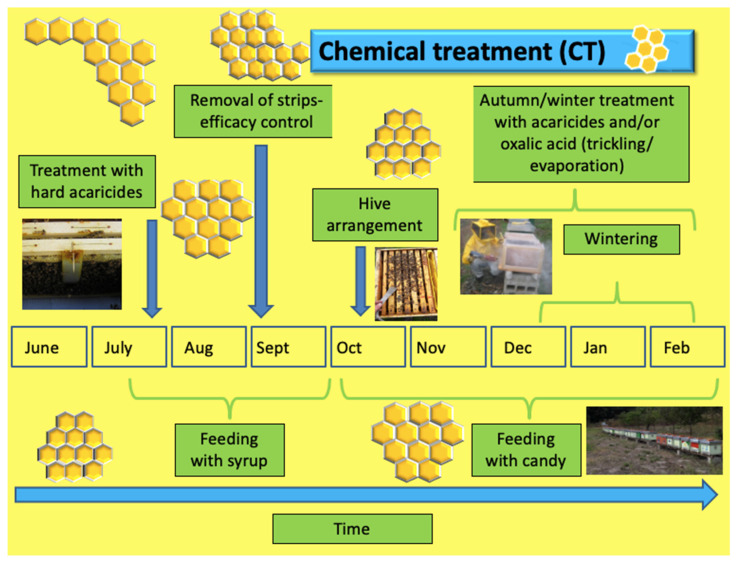
Schema of chemical treatment administration and other operations conducted in the apiary.

The farms applying thymol (THY) and chemical treatments (CT), as shown in Figure 6 and Figure 7, included the insertion of the respective strips in accordance with the label directions in July in the absence of the super. The thymol strips were changed weekly during the one-month period and removed permanently in August. In contrast, synthetic acaricide strips remained in the hive for a period ranging from 6 to 8 weeks.

During the summer period, colonies were supported by syrup-based artificial nutrition to stimulate queen laying and colony recovery. After the various *Varroa* control methods had been carried out, monitoring was conducted on the effectiveness of the operations performed. These checks mainly involved counting *Varroa* that had fallen to the bottom of the hive.

The following operations and preparations for wintering are almost identical for all the methods used. Specifically, at the beginning of autumn, an assessment of the strength of the colony and diagnosis of symptoms associated with varroosis or other diseases are performed. Then, the brood nests are arranged, the number of combs corrected and defective queens replaced. Taking advantage of the fact that the brood is absent or scarce during this period, the colonies are also treated against varroosis using various products (OA only in the case of application of biotechniques) provided by regional and national veterinary programs. If needed, syrup is supplied. Final arrangement of the nest and adjustment of the strength of the colony to the number of combs remaining for winter is carried out at the beginning of November/December.

Winter feeding in the form of candy is carried out and, in case of heavy mite infestation, treatment via sublimation of OA is repeated several times. A final check of the colonies is carried out in spring.

Via surveys conducted with the beekeepers, data regarding costs and revenue related to each step were collected and processed. The net incomes of the nine beekeeping farms are shown in Table 2.

The NI per hive, determined for the different beekeeping farms and differentiated within the same farm using several management methods, showed different values when comparisons at the inter- and intrafarm level were carried out. With regard to the TBR technique, the NI ranged from 157 to 181 EUR/hive. For CI, the values ranged from 100 to 132 EUR/hive and for QC from 106 to 155 EUR/hive. Where chemical treatment was implemented, in two out of three cases, this resulted in a lower remuneration compared to biotechniques with NIs of 88 EUR/hive (farm number 7) and 83 EUR/hive (farm number 8), whereas one farm (number 9) had a higher NI at 194 EUR/hive. 

What differences did the economic analysis carried out at farm level show in terms of NI/hive? As can be seen by the intrafarm comparisons in farm numbers 1,2 and 4, the difference in net income between TBR and CI technique was higher than that achieved between TBR and QC (Table 2). On farm number 1, a difference of 60 EUR/hive (TBR-CI) compared to 54 EUR/hive (TBR-QC) was observed, whereas for farm number 2, we calculated a difference of 42 EUR/hive compared to 35 EUR/hive. On farm number 4, TBR-CI value is equal to 48 EUR/hive, while TBR-QC corresponds to 43 EUR/hive. For farm number 3, which applied TBR and QC only, the difference in the NI was equal to 25 EUR/hive. Farm number 5, which applied TBR and thymol, shows a difference in NI equal to 73 EUR/hive, while farm number 6, using TBR alone, obtained a net income of 181 EUR/hive.

Furthermore, the analysis also showed that the two innovative techniques, TBR and then QC, were more profitable, while CT provided lower profitability and brings greater uncertainty to the economic outcome (in two out of three cases). 

## 4. Discussion

Honey bees, as well as other wild pollinators, are essential in their performance of the ecosystem service of pollination, which is fundamental to the conservation of plant biodiversity and the provision of agricultural goods [3,64]. It is therefore necessary to monitor the various threats to honey bees and beekeeping and counter their negative effects. This is particularly important because the market for bee products and the pollination of a huge number of cultivated plants have considerable economic importance that has been compromised in recent years [3,65].

In our study, beekeepers confirmed that one of the major threats is *Varroa destructor,* the mite responsible for varroosis, an external parasitic disease that attacks broods and adult honey bees [21]. This disease plagues beekeepers in their apiary and farm management. Investigations concerning the economic impact (e.g., total revenues and total expenses) and honey bee colony inventory scheme development on these farms were presented in our previous study [61,62]. Confirming previous studies [18,19,66], beekeepers also reported that global warming is extending or maintaining the presence of the brood during the winter period, and thus the spread of *Varroa* increases the risk of colony loss. As a consequence, some beekeepers from the investigated farms applied different techniques to lower the risk of *Varroa* by safeguarding the health of colonies. Like other beekeepers around the world, they looked for more sustainable and ecological solutions to maintain an acceptable level of production of honey and other products.

One *Varroa* control method is often not sufficient; control methods are commonly combined by beekeepers and may be repeatedly applied during a season [67,68]. In fact, five of the farms applied different biotechnical methods in their apiary, differentiating these techniques between beehives in order to be able to assess the best outcome and avoid the risk of getting the technique wrong on all the bee stock. On the other hand, one farm chose to apply TBR on all the apiaries to standardize work and materials. In contrast, three beekeeping farms decided to administer chemical treatments to fight *Varroa*, sometimes repeating them several times per year, which, in two out of three cases, resulted in lower revenue values than farms that used biotechniques. In terms of net income, the interfarm comparison showed that NI derived using the TBR technique was the highest in all the five case studies that applied more than one method, followed by QC and then CI. Also, the farm applying TBR as a unique technique for *Varroa* control achieved a high NI. For two case studies, the NI calculated for farms using CT was lower than for those using other methods, whereas for bee farm number 9, because it was the only family-owned farm with no salaried labor and adopting only retail sales, it showed a higher NI. From the intrafarm comparisons, TBR produced a higher NI, and the difference in NI between TBR and other techniques applied on the same farm appeared to be closely related to the survival of nuclei, which is influenced by the way in which the technique is executed, the nucleus formation period, queen fecundation, the environmental conditions and apiary management. 

In general, almost all the differences in the recorded NIs related to the different techniques could be attributed to the expenses and revenues applicable to each technique. Expenses included labor, materials (e.g., frames, cages), treatments, queens and royal cells, sugar nutrition and the fuel and travel time associated with periodic hive inspections. The main revenues were attributable to the number of nuclei produced, the variation in bee stocks (in particular for TBR), the average production of honey per hive and the sale channels used [61,62]. 

Despite the important results emerging from this study, as synthetized in Table 2, it is important to underline that the data are obtained from nine case studies, as already mentioned. Thus, it would be very important to collect further data in the future to build a larger dataset to be able to more effectively validate the results obtained from the evaluations of each biotechnique.

As demonstrated by beekeepers around the world and researchers [29,31,47,51], biotechniques are practices that entail additional expenses (supplementary hive boxes, cages, syrup, etc.), as well as a greater commitment in terms of labor hours. More recently, there have also been increases in raw material and transportation costs, which could exacerbate the expenses.

Our study has answered three questions (see Section 2). (1) Does implementation of sustainable practices on a beekeeping farm involve higher labor and material costs? The answer is yes. (2) Does use of biotechniques result in zero-residue products that can be marketed at a higher price? The answer is, again, yes, (3). Is it necessary to compromise on the health of products to save costs? The answer is no; there is no need for a trade-off. 

The adoption of biotechnical methods would appear to involve a trade-off between the profit that can be obtained and the implementation of sustainable *Varroa* control measures. However, our study showed for the first time that even in economic terms, it can be profitable for beekeeping farms to adopt biotechnical *Varroa* control measures. The beekeepers who were interviewed stated that the use of biotechniques simultaneously allowed them to increase their bee stock even for nuclei with new queens. Furthermore, the CI method applied in a large number of beehives made it possible to take advantage of genetic selection in the introduction of new queens to the colonies, which is a very important aspect considering climate change drivers. 

Our interviews showed that the greater organizational effort required for TBR and other biotechniques is balanced by monetizable factors, such as the increase in income obtained from the nuclei produced, and non-monetizable factors, such as the limitation of the emergence of resistance to acaricides and the greater resilience of the beekeeping farm in the medium to long term.

Good beekeeping practices are recommended worldwide [42], and the policies enacted by the European Commission and its Member States to finance the beekeeping sector could be exploited to adopt biotechnical methods for sustainable *Varroa* control. Both the CAP 2023–2027 [19,69] and national funds (e.g., in Italy through the measure contained in the law on the distribution of funds in support of the beekeeping sector) are crucial accessible tools for the economic support of bee farms [70]. In addition, the financing per hive supported by the National Strategic Plans 2023–2027 increases the availability of financing for farms and, consequently, can contribute to the application of *Varroa* control methods that have high management costs. This route will have a positive contribution, both by making adoption of organic production methods easier and by facilitating the application of combined solutions to fight *Varroa* and related viruses, as well as other diseases. Another significant aspect is the ability to obtain products from healthy hives which meet the expectations of the most demanding consumers [71,72] and the most evolved markets [73].

In conclusion, our study, which directly involved beekeepers, suggests that the use of biotechniques is a long-term sustainable solution for combating *Varroa* and protecting the net income of the farm. Because the brood is absent or interrupted by the application of TBR, QC, and CI, the efficacy of OA treatment is assured. Furthermore, the use of OA enables zero-residue bee products to be obtained and environmental and human health to be protected, in compliance with European and national regulations and best practice recommendations.

Biotechnical control could be complemented by other practices included in the BMP and IPM strategies, such as the use of acaricides containing naturally based active ingredients, hyperthermia and genetic selection (e.g., hygienic practices, breeding of *Varroa*-tolerant bees) [14]. Additionally, because varroosis occurs during the fall or winter, as a result of poor population health and climate change, some biotechniques such as queen caging could be carried out in late fall or winter, as already implemented by some beekeepers, to prevent colonies from continuously rearing brood with critical mite and virus infection levels.

## Figures and Tables

**Table 1:** Number of productive beehives present at the time of *Varroa* control in the apiaries and number of beehives per practice used for *Varroa* control.

Bee Farm ID	Number of Beehives in Summer	Number of TBR Beehives	Number of Beehives Subject to Other Techniques	Other Adopted Biotechniques *	Other Methods **
1	1000	200	800	QC-CI	-
2	850	100	750	QC-CI	-
3	190	50	140	QC	-
4	200	40	160	QC-CI	-
5	21	8	13	THY	-
6	160	160	-	-	
7	603	-	-	-	CT
8	1200	-	-	-	CT
9	150	-	-	-	CT

* TBR: total brood removal; QC: queen caging; CI: royal cell insertion; THY: thymol treatment. ** CT: chemical treatments. The number of treated beehives corresponds to the number of beehives present in the apiary during the summer.

**Table 2:** Net income per hive in the bee farms applying different methods for *Varroa* control.

	Bee Farm Net Income (EUR/Hive)								
*Varroa* Control Technique *	1	2	3	4	5	6	7	8	9
TBR	159.92	174.03	180.45	156.54	172.32	181.28	-	-	-
QC	106.34	138.87	155.24	113.27	-	-	-	-	-
CI	99.79	132.43	-	108.33	-	-	-	-	-
THY	-	-	-	-	99.57	-	-	-	
CT	-	-	-	-	-	-	88.41	82.81	193.65

* TBR: total brood removal; QC: queen caging; CI: royal cell insertion; THY: thymol treatment; CT: chemical treatments.

## Data Availability

The data presented in this study are available on request from the corresponding author.
